# Combined use of coenzyme Q10 and citicoline: A new possibility for patients with glaucoma

**DOI:** 10.3389/fmed.2022.1020993

**Published:** 2022-12-15

**Authors:** Alessio Martucci, Raffaele Mancino, Massimo Cesareo, Maria Dolores Pinazo-Duran, Carlo Nucci

**Affiliations:** ^1^Ophthalmology Unit, Department of Experimental Medicine, University of Rome “Tor Vergata”, Rome, Italy; ^2^Ophthalmic Research Unit “Santiago Grisolia”, Foundation for the Promotion of Health and Biomedical Research of the Valencian Community (FISABIO), Valencia, Spain; ^3^Cellular and Molecular Ophthalmobiology Group, Department of Surgery, University of Valencia, Valencia, Spain

**Keywords:** coenzyme Q10 (CoQ10), citicoline, glaucoma, neuroprotection, retinal ganglion cell death

## Abstract

Glaucoma is the leading cause of irreversible blindness worldwide. Several risk factors have been involved in the pathogenesis of the disease. By now, the main treatable risk factor is elevated intraocular pressure. Nevertheless, some patients, whose intraocular pressure is considered in the target level, still experience a progression of the disease. Glaucoma is a form of multifactorial ocular neurodegeneration with complex etiology, pathogenesis, and pathology. New evidence strongly suggests brain involvement in all aspects of this disease. This hypothesis and the need to prevent glaucomatous progression led to a growing interest in the pharmacological research of new neuroprotective, non-IOP-lowering, agents. The aim of this paper is to report evidence of the usefulness of Coenzyme Q10 and Citicoline, eventually combined, in the prevention of glaucomatous neurodegeneration.

## Introduction

Glaucoma, the leading cause of irreversible blindness worldwide, is characterized by progressive optic nerve (ON) degeneration due to retinal ganglion cells (RGCs) death. This causes characteristic ON changes and corresponding visual field defects (1–5).

Several risk factors have been involved in the pathogenesis of the disease. Understanding the molecular and cellular changes causing glaucomatous neurodegeneration includes new and path-breaking investigations on neuroinflammation and neuroprotection. Immune response, oxidative stress and gene expression are considered possible pathogenetic mechanisms of glaucoma (6–12).

Although glaucoma is a multifactorial disease, by now, the main treatable risk factor is elevated intraocular pressure (IOP). Several clinical studies reported the importance of lowering IOP in glaucomatous patients. The Ocular Hypertensive Treatment Study reported the efficacy in preventing the onset of the disease at 5 years in 50% of healthy individuals with elevated IOP (13). The Collaborative Initial Glaucoma Treatment Study observed that both surgery and IOP-lowering medications significantly reduce perimetric progression (14). Moreover, the Collaborative Normal-Tension Glaucoma Study showed that a reduction of the IOP by 30% reduced the incidence of visual field progression in a significant percentage of patients affected by normal-tension glaucoma (15). The lowering of IOP positively influences the risk of developing glaucoma and the progression of the existing disease. However, IOP alone does not explain all the risks (16). This supports the hypothesis that other risk factors independent from IOP are involved in glaucomatous degeneration.

Several studies have shown a strong correlation between visual field damage and visual disability in patients with glaucoma, even in the early stages of the disease. Visual impairment due to glaucoma affects normal daily activities required for independent living, such as driving, walking, and reading. Decreased visual functioning due to glaucoma has many disabling consequences in patients’ daily lives that, in turn, alter their quality of life (17).

New evidence strongly suggests brain involvement in all aspects of glaucoma (18). Studies using magnetic resonance imaging (MRI) have shown that the disease extends beyond the eye, altering the entire visual pathway (19, 20), indicating a connection with other neurodegenerative (17, 21–23) and mitochondrial diseases (24) as well as with disconnection syndromes (25, 26). A recent paper showed that patients with Primary Open Angle Glaucoma (POAG) exhibit a whole-brain structural reorganization that involves a variety of brain regions that take part in visual processing, motor control, and emotional/cognitive tasks. Additionally, it has been recognized a specific pattern of brain structural changes in relation to POAG clinical severity (27) that possibly justifies the glaucoma-induced functional and daily living disability (28, 29).

The need to prevent glaucomatous progression led to a growing interest in the pharmacological research of new neuroprotective, non-IOP-lowering, agents (30, 31). Citicoline and Coenzyme Q10 (CoQ10) are among the most studied and used in clinical practice for some forms of neurodegeneration. The neuromodulatory and neuroprotective properties of citicoline were extensively investigated either *in vivo* or *in vitro*. In addition, several studies supported the neuroprotective effects of CoQ10 in experimental models of ocular neurodegeneration (32).

The aim of this paper is to report evidence of the usefulness of CoQ10 and Citicoline, eventually combined, in the prevention of glaucomatous neurodegeneration.

## Coenzyme Q10

Coenzyme Q10 is also known as ubiquinone as it is a coenzyme family that is ubiquitous in animals and most bacteria. Being an electron carrier from complexes I and II to complex III, CoQ10 has a fundamental role in the production of the adenosine triphosphate (ATP), but it is also an important antioxidant that protects lipids, proteins, and DNA from oxidative stress. Due to its properties, CoQ10 has been used for a long time to treat many diseases such as Leber hereditary optic neuropathy, cerebral ischemia, Parkinson’s disease, and Huntington’s disease (33).

Coenzyme Q10 activity has been extensively studied. *In vitro*, CoQ10 prevented the activation of the optic nerve’s (ON) astrocytes induced by hydrogen peroxide. It significantly decreased two well-known processes that activate during oxidative stress: the Superoxide dismutase 2 (SOD2) and Heme oxygenase-1 (HO-1) protein expression. Hence, CoQ10 was able to prevent mitochondrial damage and the decline of ATP production (34).

Coenzyme Q10 appears an effective therapy in preventing RGCs apoptosis and loss in animal models. Intraocular administration of CoQ10 avoided RGCs death by apoptosis through the inhibition of mitochondrial depolarization by preventing the formation of the mitochondrial permeability transition pore (PTP) and reducing the glutamate increase (35). Similarly, the topical administration of CoQ10 0.1% significantly reduced staurosporine (SSP)-induced RGCs apoptosis in a rat model (36). The same effect on RGCs was reported in a model of transient ischemia where the topical administration of CoQ10 and vitamin E α–tocopherol polyethylene glycol succinate (TPGS) reduced retinal damage and prevented RGCs death possibly by inhibiting the PTP formation and cytochrome c activation. The ability of CoQ10 of reducing the accumulation of extracellular glutamate is considered one of the mechanisms underlying the protective effect on RGCs (37–39).

Moreover, in a surgically induced ocular hypertension (OHT) experimental model, Davis et al. showed a significant neuroprotective effect on RGCs using Detection of Apoptotic Retinal Cells (DARC) on a unilateral model in Adult Dark rats treated with CoQ10/TPGS micelles (40). Oral supplementation of CoQ10 was also shown to significantly increase survival of RGCs, decrease SOD-2 and HO-1 protein expression, and inactivate the astroglial and microglial cells in an animal model of OHT and in glaucomatous DBA/2J mice (41, 42). Efficacy data were not only reported in animals. In humans, the topical application of 2 drops per day of CoQ10 and vitamin E TPGS, in addition to the β-blocker monotherapy, significantly improved the visual-evoked potential (VEP) response in glaucomatous patients after 6–12 months of treatment compared to those only treated with IOP lowering medications (43).

Coenzyme Q10 bioavailability is extremely variable, and it may depend on the dosage or the delivery strategies. Achieving an optimal CoQ10 concentration is fundamental to reaching the clinical effect. Nutritional replenishment of CoQ10 requires a higher level than is available in most food. The normal level in blood is around 1 μg/ml. To increase the concentration significantly requires at least 100 mg/day which can increase the level in the blood to around 2 μg/ml or more. An increase to 2 μg/ml in the blood can be therapeutic for various conditions; this may indicate that a high blood level is needed to get CoQ10 into deficient tissues (44). Emulsified formulation and plasma lipid profiles are important factors for the absorbance of CoQ10 (38). Interestingly, a novel time-released formulation based on Miniactives^®^ showed to be safe and to increase plasma concentration of CoQ10 during the treatment (38). Consequently, it has been reported to be a promising way to deliver the molecule (45).

## Citicoline

Citicoline (cytidine-5′-diphosphocholine) is an endogenous intermediary compound in the synthesis of phospholipids’ membranes, such as phosphatidylcholine. Citicoline contributes through the multifactorial mechanism of action and intervening in several metabolic pathways, including phospholipid homeostasis, mitochondrial dynamics, as well as cholinergic and dopaminergic transmission, in the complex mechanism of visual transmission (46).

Evidence of its ability to reduce glutamate-mediated excitotoxicity and oxidative stress by boosting neurotrophin levels and supporting mitochondrial activity endorsed the use of citicoline in neurodegenerative diseases (47).

Oral citicoline has been reported to increase the release of dopamine and norepinephrine and its efficacy has been proved in several neurodegenerative diseases such as Alzheimer’s disease, Parkinson’s disease, as well as in ischemic and traumatic brain injury. The possible neuro-enhancing effect, possibly due to the dopamine increase, justifies the improvement of visual field and electrophysiological test results obtained in glaucomatous patients (46, 47).

Oral citicoline is usually well absorbed and, after its transformation into choline and cytidine in the intestinal wall and liver, it crosses the blood-brain barrier. Hence, supplies the metabolic precursors of phospholipids and participates in the synthetic pathways of nucleic acids, proteins, phosphatidylcholine, sphingomyelin, cardiolipin, and acetylcholine, the main neurotransmitter of the cholinergic system which modulates visual processes. Beyond this, citicoline acts as a rescue recourse for cellular membrane components (46, 48).

Literature suggests that citicoline can reduce the pro-apoptotic effects and synaptic loss in neural tissues. Citicoline’s ability in maintaining the proper acetylcholine metabolism and the proper levels of sphingomyelin make it a good candidate for supporting the RGCs’ axonal function and consequently enhancing their survival (48). Additionally, this antiapoptotic effect seems to be also connected to the activity of the mitochondrial-dependent cell death mechanism. Indeed, citicoline prevents ischemia-induced tissue increase of free fatty acids and decreases infarct volume and brain oedema (49, 50).

Studies using optical coherence tomography showed that citicoline prevents the loss in the average retinal nerve fiber layer in glaucoma patients (47). Therefore, citicoline may have a significant impact on slowing glaucoma progression, suggesting a potential neuroprotective effect (47, 51).

Overall, these data make citicoline a candidate for the treatment of glaucomatous neurodegeneration.

## Combined use of CoQ10 and citicoline: A new strategy in glaucoma treatment

**FIGURE 1:**
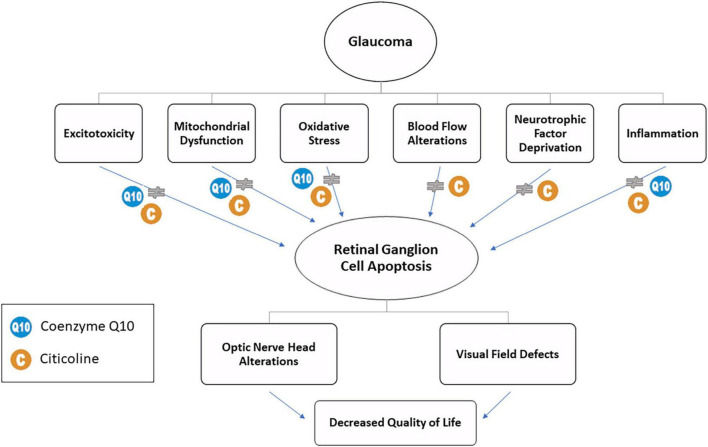
The involvement of citicoline and CoQ10 in glaucoma.

Glaucoma is an extremely complex disease; therefore, it is now evident that the treatment should be targeted at different aspects of the disease, possibly by combining several molecules. However, prescribing several medications may affect the quality of life of the patients who must follow complex, and sometimes, expensive treatments. To overcome these difficulties, new combined products have been produced (Figure 1).

**FIGURE 2:**
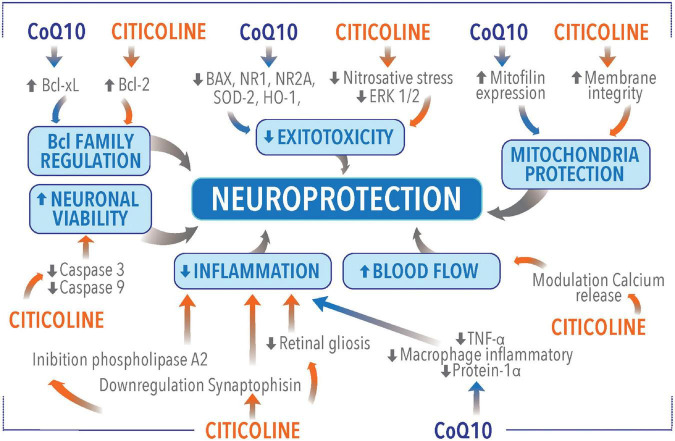
Neuroprotective molecular mechanisms of coenzyme Q10 (CoQ10) and citicoline.

Nowadays, CoQ10 and Citicoline are among the most used molecules in glaucoma for neuroprotection. The two molecules act on different pathways leading to glaucomatous neurodegeneration. As a consequence, it is possible to speculate that CoQ10 and citicoline may have a complementary or a synergic effect as they act on different pathogenetic targets in glaucoma (Figure 2 and Table 1).

**TABLE 1:** Principal studies describing neuroprotective molecular mechanisms of coenzyme Q10 (CoQ10) and citicoline.

Mechanism	References	Outcomes
Bcl family proteins regulation	(52)	Neuroprotection provided by citicoline is due to an increased retinal expression of the apoptotic regulating protein Bcl-2 possibly mimicking brain-derived neurotrophic factor.
	(41)	CoQ10 promoted RGC survival in glaucomatous DBA/2J mice. CoQ10 significantly decreased Bax protein expression, that is a proapoptotic member of the Bcl-2 family, essential in many pathways of apoptosis.
Excitotoxicity regulation	(53)	Morphometric analysis showed a significant reduction in inner nuclear and inner plexiform layers thicknesses and ganglion cell loss after kainic acid injection, but the rate of thinning in retinal layers was reduced after citicoline treatment.
	(54)	Citicoline has a neuroprotective effect on retinal damage due to kainic acid-induced neurotoxicity.
	(42)	CoQ10 promotes RGC survival by inhibiting oxidative stress, glutamate excitotoxicity, and activation of the Bax and Bad–mediated apoptotic pathway and by preserving mtDNA content and Tfam/OXPHOS complex IV protein expression in glaucomatous DBA/2J mice.
Caspases regulation	(52)	Citicoline can reduce the expression of active forms of caspases-9 and-3 regulating apoptosis.
Effect on mitochondria	(52)	Citicoline increase the availability of nucleotides essential for the synthesis of membrane phospholipids and enhances bioenergetics and phospholipid membrane turnover in the brain.
	(34)	CoQ10 promote mitofilin protein expression, providing protection to the mitochondria, and ultimately OXPHOS capacity against oxidative stress.
	(44)	CoQ10 has bioenergetic properties. CoQ10 reduction/oxidation cycles transfer protons across the membrane forming a proton gradient essential to producing ATP.
Neurotransmitters system stimulation	(56)	Citicoline reinforces dopaminergic transmission in the retina
Membrane integrity maintenance	(46)	CDP-choline is a precursor of glycerophospholipid phosphatidylcholine which is an essential phospholipid for the maintenance of intracellular and extracellular membranes of eukaryotic organisms; these molecules are of particular relevance in surveying neuron homeostasis and functionality, by serving membranes turnover, synaptic plasticity, and neurotransmission.
	(60)	Citicoline leads to the formation of phosphatidylcholine, which is an important component of neuronal membranes and is imperative to the membrane integrity of the retinal ganglion cells.
Anti-inflammatory properties	(46)	Citicoline was found to be protective in transient cerebral ischemia through the inhibition of phospholipase A2.
	(62)	CoQ10 induced a significant reduction of TNF-α level, lessening the production of pro-inflammatory cytokines and lowering the production of macrophage inflammatory protein-1 alpha.
Cerebral blood flow improvement	(60)	Citicoline inducing calcium release from endothelial cells improves endothelial function leading to improved microvasculature and better blood flow.
	(64)	Prescription of citicoline for treatment of acute ischemic stroke is associated with hemodynamic changes in cerebral arteries.
	(63)	Citicoline is well tolerated and improves cognitive performance, cerebral blood perfusion and the brain bioelectrical activity pattern in Alzheimer’s disease.
Mutual beneficial effect on molecules metabolism	(65)	Ubiquinone is necessary for the L-3-glycerophosphate oxidase of pig brain mitochondria.
	(58)	Choline oxidization is restored by the addition of ubiquinone-2 or ubiquinone-10 to the oxidase assay medium. CoQ10 was found to increase the choline oxidase activity in ubiquinone-depleted mitochondria.
	(66)	Solubilized choline dehydrogenase is capable to reduce ubiquinone.
	(68)	CoQ10 showed a neuroprotective activity, in the case of choline depletion, by inhibiting the release of glutamate in rat cerebrocortical nerve terminals.

Interestingly, a recent paper highlighted the complete biocompatibility of citicoline, CoQ10, and vitamin B3 tested using a viability MTT test. When the compounds were co-administered at the highest concentration tolerated by the cells (10 μM), in basal conditions, the biocompatibility was preserved, and the cell viability (hypothalamic HypoE22 cells), in all pharmacological treatments, was always > 70% compared to the untreated cells (32).

### Effect on Bcl family proteins regulation

An experimental model of progressive degeneration of RGCs, induced by partial ON crush, produced a selective loss of RGCs, similar to glaucoma. Neurons in fact, although their axons were not acutely damaged, degenerated due to pro-apoptotic environmental conditions produced by the initial injury. The administration of citicoline was effective in rescuing RGCs possibly increasing retinal expression of the apoptotic regulating protein Bcl-2 as well as acting as a BDNF mimic (52).

In a similar experimental condition, CoQ10 promoted RGCs survival by significantly inducing Bcl-xL protein expression, a member of the BCL-2 family that exert cytoprotective and anti-apoptotic functions via several mechanisms. Bcl-xL avoids the generation of proapoptotic cytosolic Ca2+ waves, segregates a cytosolic pool of the pro-apoptotic transcription factor p53 and binds to the voltage-dependent anion channel 1, thereby inhibiting the mitochondrial permeability transition–dependent apoptotic pathway. Bcl-xL also regulates mitochondrial ATP synthesis, protein acetylation, autophagy, and mitosis (42).

### Effect on excitotoxicity

Citicoline and CoQ10 have been reported to counteract excitotoxicity. The neuroprotective effect of citicoline was reported in an animal model of kainic acid (KA)-induced retinal damage (53, 54). All these studies showed that citicoline caused a significant reduction of KA-induced damage in the retinas of treated animals apparently counteracting nitrosative stress and decreasing extracellular signal-regulated kinases (ERK)1/2 activation caused by KA (55).

Similarly, CoQ10 reduces glutamate excitotoxicity and oxidative stress-mediated RGCs degeneration by preventing mitochondrial alterations in the retina in animal models. CoQ10 supports RGC survival, protected the axons in the optic nerve head (ONH), and reduced astroglial activation by decreasing glial fibrillary acidic protein expression in the retina and ONH. CoQ10 prevented the upregulation of NR1 and NR2A, Superoxide dismutase 2 and heme oxygenase 1 protein expression, and significantly prevented apoptosis by decreasing Bax protein expression or by increasing pBad protein expression. More importantly, CoQ10 preserved mtDNA content and Mitochondrial transcription factor A (Tfam)/oxidative phosphorylation (OXPHOS) complex IV protein expression in the retina of glaucomatous DBA/2J mice (42).

### Effect on caspases

Studies on animals showed that citicoline can rescue damaged RGCs through an anti-apoptotic effect and can support neurite regeneration of damaged RGCs. Similarly, to Brain-derived neurotrophic factor (BDNF) and NT-4, citicoline can reduce, in an animal model, retinal neuronal apoptosis and stimulate neurites’ regeneration by lowering the expression of active forms of caspases-9 and-3 (52).

### Effect on mitochondria

Animal models showed that citicoline can reverse some aging mitochondrial processes, possibly due to its ability to increase the availability of nucleotides essential for the synthesis of membrane phospholipids, phosphatidylserine and phosphatidylethanolamine, and/or to enhance brain energy metabolism (52).

Similarly, in a resonance magnetic study on volunteer humans, citicoline significantly increased the phosphocreatine, the ATP, the ratio of phosphocreatine to inorganic phosphate, and induced improvements in membrane phospholipids. This is indicative that citicoline enhances bioenergetics and phospholipid membrane turnover in the brain (52).

Mitofilin, the principal mitochondrial inner membrane protein which plays a crucial role in the preservation of mitochondrial cristae morphology, reduces in the condition of oxidative stress. In this condition CoQ10 showed partial preservation of mitochondrial morphology, increased mitochondrial numbers and mitochondrial volume density. This is possibly due to CoQ10 ability to promote mitofilin protein expression, providing protection to the mitochondria and ultimately OXPHOS capacity against oxidative stress (34).

In addition, CoQ10 plays a vital role in ATP production. In mitochondria and lysosomes, CoQ10 goes through reduction/oxidation cycles that transfer protons across the membrane forming a proton gradient essential to producing ATP (44). Thus supporting the bioenergetic role of CoQ10.

### Effect on neurotransmitters system

As seen, citicoline not only exerts a neuroprotective effect but also boosts the synthesis of dopamine, acetylcholine, noradrenaline and serotonin. Studies reported that after citicoline administration retinal dopamine levels significantly increased thus possibly in part justifying the improvement of visual function in glaucomatous patients in terms of visual field and electrophysiological tests results (56).

### Effect on membrane integrity

One additional target on which citicoline may act is remyelination. Disruption of the axonal membranes in glaucoma has been previously described since the early stages of the disease (19, 24, 57). Citicoline, is a precursor for phosphatidylcholine, phosphatidylethanolamine, sphingomyelin and cardiolipin, which are fundamental structural and functional components of cell membranes that ensure the correct enzymatic viability for the transport of substances across the membrane and are essential in signal transduction. Being a protagonist in maintaining membrane integrity, citicoline also plays a pivotal role in counteracting axonal degeneration in glaucoma (46, 58–60).

### Effect on inflammation

Citicoline has been shown to have a positive protective effect on inflammatory diseases (61).

Citicoline has been shown to counteract the pathological downregulation of synaptophysin in the retina, restoring its anti-inflammatory properties. Moreover, citicoline is able to reduce retinal reactive gliosis, prevent apoptosis of the entire retinal components, such as photoreceptors, bipolar cells, and RGCs.

Similarly, the administration of citicoline resulted to be protective in transient cerebral ischemia through the inhibition of phospholipase A2 (PLA2), with a consequent reduction of tissue inflammation and redox imbalance (46).

Coenzyme Q10 claims some anti-inflammatory properties as well. A recent systematic review and meta-analysis reported that improving the serum level of CoQ10 induced a significant reduction of TNF-α level in the CoQ10 supplementation group compared with placebo. This may be due to the potential role of CoQ10 in lessening the production of pro-inflammatory cytokines by preventing NF-κB gene expression, reducing miR-146a and IL-1 receptor associated kinase modulation. CoQ10 may also act by lowering the production of macrophage inflammatory protein-1 alpha (62).

### Effect on cerebral blood flow

Citicoline was shown to improve cerebral blood flow and velocities compared to placebo. This is possibly due to the effect on calcium release from endothelial cells that regulate nitric oxide synthesis consequently enhancing endothelial function. The proper functioning of the microvasculature and the right tissue perfusion is crucial for neuronal viability. In this context, restoring endothelial dysfunction leads to healthier microvasculature and improved blood flow, avoiding neuronal apoptosis (60, 63, 64).

### Mutual beneficial effect on molecules metabolism

Data on literature, suggests that the combined use of molecules may be also beneficial for the metabolism of the molecules themselves. Barrett and Dawson (58) reported that rat liver mitochondria treated extensively with n-pentane are incapable of oxidizing choline. Choline oxidization is restored by the addition of ubiquinone-2 or ubiquinone-10 to the oxidase assay medium. The necessity for ubiquinone of the L-3-glycerophosphate oxidase of pig brain mitochondria has been also previously confirmed (65). Ubiquinone is also fundamental for Nicotinamide adenine dinucleotide (NADH) and succinate oxidase (58). Previous studies showed that solubilized choline dehydrogenase is capable to reduce ubiquinone-6 and that, in mitochondria incubated with choline until the anaerobic state was achieved, endogenous ubiquinone was reduced (66). The choline dehydrogenase is a respiratory-chain-linked enzyme that supplies electrons into the respiratory chain. It can use ubiquinone-6 as an electron acceptor once the enzyme has been solubilized and interacts with the chain in such a manner as to suggest that the possibility of reversed electron transport from choline to NAD+ strongly implicates a requirement of the choline oxidase system for ubiquinone. Remarkably, Barrett and Dawson (58) observed that most of the choline oxidase activity was reduced because of ubiquinone depletion. In this regard, CoQ10 was found to increase the choline oxidase activity in ubiquinone-depleted mitochondria, thus suggesting the importance of the presence of good levels of plasmatic choline and CoQ10 for energetic production (58).

Qu et al. (67) showed that CoQ10 decreases in the human retina with aging. In people aged over 80 retinal CoQ10 levels declined by approximately 40% compared to people under 30. This may have two main consequences: a decrease in antioxidant ability and a decrease in the rate of ATP synthesis in the retina. This would make the RGCs more vulnerable to pro-apoptotic insults.

Moreover, a study on an experimental animal model of non-alcoholic steatohepatitis (NASH) in albino rats induced by a methionine and choline-deficient (MCD) diet showed a significant increase in the brain contents of ammonia and NOx. These substances were significantly reduced by treatment with CoQ10. Concomitantly the brain-derived neurotrophic factor content, which was reduced by the diet, increased. Overall, CoQ10 showed a neuroprotective activity, in the case of choline depletion, by inhibiting the release of glutamate in rat cerebrocortical nerve terminals (68).

## Conclusion

The greater efficacy of the fixed combination over the single components could therefore depend on the fact that each molecule exerts, at least in part, their activity on mitochondria (32). CoQ10 acts as an electron accepter from mitochondrial complexes I and II, thus increasing the energetic rate of cells. Additionally, citicoline maintains proper levels of cardiolipin and sphingomyelin in the cellular and axon membranes and stimulates cardiolipin production within the mitochondrial membranes. Cardiolipin is essential for the optimal activity of the enzyme complexes of the electron transport chain and for ATP production (32).

Overall, these data suggest the possible usefulness of the combined use of citicoline and CoQ10 both in terms of a putative synergistic effect and in terms of combined action on the different pathogenetic targets causing the onset and progression of glaucoma. Using combined treatment may downregulate more pro-apoptotic pathways as well as it may boost the effect on one or more pathways on which the different molecules act. In addition, it may increase patients’ compliance reducing the burden of administering several medications, simplifying treatment.

## Author contributions

All authors listed have made a substantial, direct, and intellectual contribution to the work, and approved it for publication.

## References

[1] Lin SC Singh K Jampel HD Hodapp EA Smith SD Francis BA Optic nerve head and retinal nerve fiber layer analysis: a report by the American academy of ophthalmology. *Ophthalmology.* (2007) 114:1937–49. 10.1016/j.ophtha.2007.07.005 17908595 PMC3780976

[2] Erb C Göbel K. Functional glaucoma diagnosis. *Ophthalmologe.* (2009) 106:375–85. 19343353 10.1007/s00347-008-1817-9

[3] Wu Z Medeiros FA. Recent developments in visual field testing for glaucoma. *Curr Opin Ophthalmol.* (2018) 29:141–6. 29256895 10.1097/ICU.0000000000000461

[4] European Glaucoma Society. *Terminology and guidelines for glaucoma.* 5th ed. (2020). Available online at: https://www.eugs.org/eng/egs_guidelines_reg.asp (accessed October 22, 2022). 10.1136/bjophthalmol-2021-egsguidelines 34675001

[5] Nucci C Martucci A Cesareo M Garcia F Morrone LA Russo R Links among glaucoma, neurodegenerative, and vascular diseases of the central nervous system. *Prog Brain Res.* (2015) 221:49–65. 10.1016/bs.pbr.2015.04.010 26518072

[6] Raga-Cervera J Bolarin JM Millan JM Garcia-Medina JJ Pedrola L Abellán-Abenza J miRNAs and genes involved in the interplay between ocular hypertension and primary open-angle glaucoma. Oxidative stress, inflammation, and apoptosis networks. *J Clin Med.* (2021) 10:2227. 10.3390/jcm10112227 34063878 PMC8196557

[7] Pinazo-Durán MD Zanón-Moreno V Gallego-Pinazo R García-Medina JJ. Oxidative stress and mitochondrial failure in the pathogenesis of glaucoma neurodegeneration. *Prog Brain Res.* (2015) 220:127–53. 10.1016/bs.pbr.2015.06.001 26497788

[8] Pinazo-Durán MD Shoaie-Nia K Zanon-Moreno V Sanz-Gonzalez SM Del Castillo JB Garcia-Medina JJ. Strategies to reduce oxidative stress in glaucoma patients. *Curr Neuropharmacol.* (2018) 16:903–18. 10.2174/1570159X15666170705101910 28677495 PMC6120109

[9] Pinazo-Durán MD García-Medina JJ Bolarín JM Sanz-González SM Valero-Vello M Abellán-Abenza J Computational analysis of clinical and molecular markers and new theranostic possibilities in primary open-angle glaucoma. *J Clin Med.* (2020) 9:3032. 10.3390/jcm9093032 32967086 PMC7564865

[10] Pinazo-Durán MD Muñoz-Negrete FJ Sanz-González SM Benítez-Del-Castillo J Giménez-Gómez R Valero-Velló M The role of neuroinflammation in the pathogenesis of glaucoma neurodegeneration. *Prog Brain Res.* (2020) 256:99–124. 10.1016/bs.pbr.2020.07.004 32958217

[11] Zanon-Moreno V Marco-Ventura P Lleo-Perez A Pons-Vazquez S Garcia-Medina JJ Vinuesa-Silva I Oxidative stress in primary open-angle glaucoma. *J Glaucoma.* (2008) 17:263–8. 18552610 10.1097/IJG.0b013e31815c3a7f

[12] Pinazo-Durán MD Zanón-Moreno V García-Medina JJ Gallego-Pinazo R. Evaluation of presumptive biomarkers of oxidative stress, immune response and apoptosis in primary open-angle glaucoma. *Curr Opin Pharmacol.* (2013) 13:98–107. 10.1016/j.coph.2012.10.007 23142105

[13] Kass MA Heuer DK Higginbotham EJ Johnson CA Keltner JL Miller JP The ocular hypertension treatment study: a randomized trial determines that topical ocular hypotensive medication delays or prevents the onset of primary open-angle glaucoma. *Arch Ophthalmol.* (2002) 120:701–13. 10.1001/archopht.120.6.701 12049574

[14] Lichter PR Musch DC Gillespie BW Guire KE Janz NK Wren PA Interim clinical outcomes in the collaborative initial glaucoma treatment study comparing initial treatment randomized to medications or surgery. *Ophthalmology.* (2001) 108:1943–53. 10.1016/s0161-6420(01)00873-9 11713061

[15] The Collaborative Normal Tension Glaucoma Study Group. Comparison of glaucomatous progression between untreated patients with normal-tension glaucoma and patients with therapeutically reduced intraocular pressures. *Am J Ophthalmol.* (1998) 126:487–97. 10.1016/s0002-9394(98)00223-2 9780093

[16] Jayaram H. Intraocular pressure reduction in glaucoma: does every mmHg count? *Taiwan J Phthalmol.* (2020) 10:255–8. 10.4103/tjo.tjo_63_20 33437597 PMC7787090

[17] Cesareo M Ciuffoletti E Ricci F Missiroli F Giuliano MA Mancino R Visual disability and quality of life in glaucoma patients. *Prog Brain Res.* (2015) 221:359–74. 10.1016/bs.pbr.2015.07.003 26518087

[18] Faiq MA Dada R Kumar A Saluja D Dada T. Brain: the potential diagnostic and therapeutic target for glaucoma. *CNS Neurol Disord Drug Targets.* (2016) 15:839–44. 26996164 10.2174/1871527315666160321111522

[19] Bolacchi F Garaci FG Martucci A Meschini A Fornari M Marziali S Differences between proximal versus distal intraorbital optic nerve diffusion tensor magnetic resonance imaging properties in glaucoma patients. *Invest Ophthalmol Vis Sci.* (2012) 53:4191–6. 10.1167/iovs.11-9345 22570349

[20] Nucci C Martucci A Cesareo M Mancino R Russo R Bagetta G Brain involvement in glaucoma: advanced neuroimaging for understanding and monitoring a new target for therapy. *Curr Opin Pharmacol.* (2013) 13:128–33. 10.1016/j.coph.2012.08.004 22981808

[21] Mancino R Martucci A Cesareo M Giannini C Corasaniti MT Bagetta G Glaucoma and alzheimer disease: one age-related neurodegenerative disease of the brain. *Curr Neuropharmacol.* (2018) 16:971–7. 10.2174/1570159X16666171206144045 29210654 PMC6120118

[22] Nucci C Martucci A Martorana A Sancesario GM Cerulli L. Glaucoma progression associated with altered cerebral spinal fluid levels of amyloid beta and tau proteins. *Clin Exp Ophthalmol.* (2011) 39:279–81. 10.1111/j.1442-9071.2010.02452.x 20973903

[23] Martucci A Picchi E Di Giuliano F Pocobelli G Mancino R Toschi N Imaging biomarkers for Alzheimer’s disease and glaucoma: current and future practices. *Curr Opin Pharmacol.* (2022) 62:137–44. 10.1016/j.coph.2021.12.003 34995895

[24] Nucci C Martucci A Mancino R Cerulli L. Glaucoma progression associated with Leber’s hereditary optic neuropathy. *Int Ophthalmol.* (2013) 33:75–7. 10.1007/s10792-012-9623-4 22983441

[25] Minosse S Floris R Nucci C Toschi N Garaci F Martucci A Disruption of brain network organization in primary open angle glaucoma. *Annu Int Conf IEEE Eng Med Biol Soc.* (2019) 2019:4338–41. 10.1109/EMBC.2019.8857290 31946828

[26] Nucci C Garaci F Altobelli S Di Ciò F Martucci A Aiello F Diffusional kurtosis imaging of white matter degeneration in glaucoma. *J Clin Med.* (2020) 9:3122. 10.3390/jcm9103122 32992559 PMC7600134

[27] Di Ciò F Garaci F Minosse S Passamonti L Martucci A Lanzafame S Reorganization of the structural connectome in primary open angle Glaucoma. *Neuroimage Clin.* (2020) 28:102419. 10.1016/j.nicl.2020.102419 33032067 PMC7552094

[28] Cesareo M Martucci A Ciuffoletti E Mancino R Cerulli A Sorge RP Association between Alzheimer’s disease and glaucoma: a study based on heidelberg retinal tomography and frequency doubling technology perimetry. *Front Neurosci.* (2015) 9:479. 10.3389/fnins.2015.00479 26733792 PMC4683203

[29] Martucci A Cesareo M Toschi N Garaci F Bagetta G Nucci C. Brain networks reorganization and functional disability in glaucoma. *Prog Brain Res.* (2020) 257:65–76. 10.1016/bs.pbr.2020.07.007 32988473

[30] Nucci C Russo R Martucci A Giannini C Garaci F Floris R New strategies for neuroprotection in glaucoma, a disease that affects the central nervous system. *Eur J Pharmacol.* (2016) 787:119–26. 10.1016/j.ejphar.2016.04.030 27089818

[31] Nucci C Martucci A Giannini C Morrone LA Bagetta G Mancino R. Neuroprotective agents in the management of glaucoma. *Eye.* (2018) 32:938–45. 10.1038/s41433-018-0050-2 29472700 PMC5944652

[32] Mastropasqua L Agnifili L Ferrante C Sacchi M Figus M Rossi GCM Citicoline/coenzyme Q10/vitamin B3 fixed combination exerts synergistic protective effects on neuronal cells exposed to oxidative stress. *Nutrients.* (2022) 14:2963. 10.3390/nu14142963 35889920 PMC9316190

[33] Zhang X Tohari AM Marcheggiani F Zhou X Reilly J Tiano L Therapeutic potential of co-enzyme Q10 in retinal diseases. *Curr Med Chem.* (2017) 24:4329–39. 10.2174/0929867324666170801100516 28762311

[34] Noh YH Kim KY Shim MS Choi SH Choi S Ellisman MH Inhibition of oxidative stress by coenzyme Q10 increases mitochondrial mass and improves bioenergetic function in optic nerve head astrocytes. *Cell Death Dis.* (2013) 4:e820. 10.1038/cddis.2013.341 24091663 PMC3824651

[35] Nucci C Tartaglione R Cerulli A Mancino R Spanò A Cavaliere F Retinal damage caused by high intraocular pressure induced transient ischemia is prevented by coenzyme Q10 in rat. *Int Rev Neurobiol.* (2007) 82:397–406. 10.1016/S0074-7742(07)82022-8 17678974

[36] Guo L Cordeiro MF. Assessment of neuroprotection in the retina with DARC. *Prog Brain Res.* (2008) 173:437–50. 10.1016/S0079-6123(08)01130-8 18929126 PMC2603274

[37] Russo R Cavaliere F Rombolà L Gliozzi M Cerulli A Nucci C Rational basis for the development of coenzyme Q10 as a neurotherapeutic agent for retinal protection. *Prog Brain Res.* (2008) 173:575–82. 10.1016/S0079-6123(08)01139-4 18929135

[38] Martucci A Reurean-Pintilei D Manole A. Bioavailability and sustained plasma concentrations of CoQ10 in healthy volunteers by a novel oral timed-release preparation. *Nutrients.* (2019) 11:527. 10.3390/nu11030527 30823449 PMC6471387

[39] Martucci A Nucci C. Evidence on neuroprotective properties of coenzyme Q10 in the treatment of glaucoma. *Neural Regen Res.* (2019) 14:197–200. 10.4103/1673-5374.244781 30530997 PMC6301180

[40] Davis BM Tian K Pahlitzsch M Brenton J Ravindran N Butt G Topical Coenzyme Q10 demonstrates mitochondrial-mediated neuroprotection in a rodent model of ocular hypertension. *Mitochondrion.* (2017) 36:114–23. 10.1016/j.mito.2017.05.010 28549843 PMC5645575

[41] Lee D Kim KY Shim MS Kim SY Ellisman MH Weinreb RN Coenzyme Q10 ameliorates oxidative stress and prevents mitochondrial alteration in ischemic retinal injury. *Apoptosis.* (2014) 19:603–14. 10.1007/s10495-013-0956-x 24337820 PMC3938850

[42] Lee D Shim MS Kim KY Noh YH Kim H Kim SY Coenzyme Q10 inhibits glutamate excitotoxicity and oxidative stress-mediated mitochondrial alteration in a mouse model of glaucoma. *Invest Ophthalmol Vis Sci.* (2014) 55:993–1005. 10.1167/iovs.13-12564 24458150 PMC3929080

[43] Parisi V Centofanti M Gandolfi S Marangoni D Rossetti L Tanga L Effects of coenzyme Q10 in conjunction with vitamin E on retinal-evoked and cortical-evoked responses in patients with open-angle glaucoma. *J Glaucoma.* (2014) 23:391–404. 10.1097/IJG.0b013e318279b836 25079307

[44] Crane FL. Biochemical functions of coenzyme Q10. *J Am Coll Nutr.* (2001) 20:591–8. 10.1080/07315724.2001.10719063 11771674

[45] Pravst I Rodríguez Aguilera JC Cortes Rodriguez AB Jazbar J Locatelli I Hristov H Comparative bioavailability of different coenzyme q10 formulations in healthy elderly individuals. *Nutrients.* (2020) 12:784. 10.3390/nu12030784 32188111 PMC7146408

[46] Oddone F Rossetti L Parravano M Sbardella D Coletta M Ziccardi L Citicoline in ophthalmological neurodegenerative disease: a comprehensive review. *Pharmaceuticals.* (2021) 14:281. 10.3390/ph14030281 33804675 PMC8003774

[47] Sahin AK Kapti HB Uzun A. Effect of oral citicoline therapy on retinal nerve fiber layer and ganglion cell-inner plexiform layer in patients with primary open angle glaucoma. *Int J Ophthalmol.* (2022) 15:483–8. 10.18240/ijo.2022.03.17 35310047 PMC8907038

[48] Skopiński P Radomska-Leśniewska DM Izdebska J Kamińska A Kupis M Kubiak AJ New perspectives of immunomodulation and neuroprotection in glaucoma. *Cent Eur J Immunol.* (2021) 46:105–10. 10.5114/ceji.2021.104329 33897291 PMC8056344

[49] Grieb P Rejdak R. Pharmacodynamics of citicoline relevant to the treatment of glaucoma. *J Neurosci Res.* (2002) 67:143–8. 10.1002/jnr.10129 11782957

[50] Secades JJ. Citicoline: pharmacological and clinical review, 2016 update. *Rev Neurol.* (2016) 63:1–73. 28417449

[51] Lanza M Gironi Carnevale UA Mele L Bifani Sconocchia M Bartollino S Costagliola C. Morphological and functional evaluation of oral citicoline therapy in chronic open-angle glaucoma patients: a pilot study with a 2-year follow-up. *Front Pharmacol.* (2019) 26:1117. 10.3389/fphar.2019.01117 31611797 PMC6775502

[52] Grieb P Jünemann A Rekas M Rejdak R. Citicoline: a food beneficial for patients suffering from or threated with glaucoma. *Front Aging Neurosci.* (2016) 8:73. 10.3389/fnagi.2016.00073 27092075 PMC4824764

[53] Han YS Chung IY Park JM Yu JM. Neuroprotective effect of citicoline on retinal cell damage induced by kainic acid in rats. *Korean J Ophthalmol.* (2005) 19:219–26. 10.3341/kjo.2005.19.3.219 16209285

[54] Park CH Kim YS Noh HS Cheon EW Yang YA Yoo JM Neuroprotective effect of citicoline against KA-induced neurotoxicity the rat retina. *Exp Eye Res.* (2005) 81:350–8. 10.1016/j.exer.2005.02.007 16129102

[55] Park CH Kim YS Cheon EW Noh HS Cho CH Chung IY Action of citicoline on rat retinal expression of extracellular-signal-regulated kinase (ERK1/2). *Brain Res.* (2006) 1081:203–10. 10.1016/j.brainres.2005.12.128 16696125

[56] Rejdak R Toczołowski J Solski J Duma D Grieb P. Citicoline treatment increases retinal dopamine content in rabbits. *Ophthalmic Res.* (2002) 34:146–9. 10.1159/000063658 12097797

[57] Michelson G Engelhorn T Wärntges S El Rafei A Hornegger J Doerfler A. DTI parameters of axonal integrity and demyelination of the optic radiation correlate with glaucoma indices. *Graefes Arch Clin Exp Ophthalmol.* (2013) 251:243–53. 10.1007/s00417-011-1887-2 22366916

[58] Barrett MC Dawson AP. Essentiality of ubiquinone for choline oxidation in rat liver mitochondria. *Biochem J.* (1975) 148:595–7. 10.1042/bj1480595 1200993 PMC1165580

[59] Osborne NN Wood JP Chidlow G Bae JH Melena J Nash MS. Ganglion cell death in glaucoma: what do we really know? *Br J Ophthalmol.* (1999) 83:980–6. 10.1136/bjo.83.8.980 10413706 PMC1723166

[60] Faiq MA Wollstein G Schuman JS Chan KC. Cholinergic nervous system and glaucoma: from basic science to clinical applications. *Prog Retin Eye Res.* (2019) 72:100767. 10.1016/j.preteyeres.2019.06.003 31242454 PMC6739176

[61] Ek RO Serter M Ergin K Cecen S Unsal C Yildiz Y Protective effects of citicoline on TNBS-induced experimental colitis in rats. *Int J Clin Exp Med.* (2014) 7:989–97. 24955172 PMC4057851

[62] Zhai J Bo Y Lu Y Liu C Zhang L. Effects of coenzyme Q10 on markers of inflammation: a systematic review and meta-analysis. *PLoS One.* (2017) 12:e0170172. 10.1371/journal.pone.0170172 28125601 PMC5268485

[63] Alvarez XA Mouzo R Pichel V Pérez P Laredo M Fernández-Novoa L Double-blind placebo-controlled study with citicoline in APOE genotyped Alzheimer’s disease patients. Effects on cognitive performance, brain bioelectrical activity and cerebral perfusion. *Methods Find Exp Clin Pharmacol.* (1999) 21:633–44. 10669911

[64] Seifaddini R Moghadam AH Iranmanesh F Arvan H Naghibzadeh-Tahami A. The effects of citicoline on cerebrovascular hemodynamic status in ischemic stroke patients. *J Kerman Univ Med Sci.* (2017) 24:480–6.

[65] Salach J Bednarz J. Essentiality of coenzyme Q for the oxidation of α-glycerophosphate by pig brain mitochondria. *Arch Biochem Biophys.* (1973) 157:133–44. 10.1016/0003-9861(73)90398-6 4146143

[66] Drabikowska AK Szarkowska L. The reduction of ubiquinone in rat liver mitochondria associated with the oxidation of choline. *Acta Biochim Pol.* (1965) 12:387–94. 5854014

[67] Qu J Kaufman Y Washington I. Coenzyme Q10 in the human retina. *Invest Ophthalmol Vis Sci.* (2009) 50:1814–8. 10.1167/iovs.08-2656 19060288

[68] Saleh DO Ahmed RF Amin MM. Modulatory role of Co-enzyme Q10 on methionine and choline deficient diet-induced non-alcoholic steatohepatitis (NASH) in albino rats. *Appl Physiol Nutr Metab.* (2017) 42:243–9. 10.1139/apnm-2016-0320 28177750

